# Thermo-Mechanical Performance of Polylactide Composites Reinforced with Alkali-Treated Bamboo Fibers

**DOI:** 10.3390/polym10040401

**Published:** 2018-04-04

**Authors:** Fang Wang, Shujue Zhou, Mengqing Yang, Zhiqian Chen, Siyan Ran

**Affiliations:** 1 Faculty of Materials and Energy, Southwest University, Chongqing 400715, China; zsj1119@email.swu.edu.cn (S.Z.); ymq123@email.swu.edu.cn (M.Y.); chen_zq@swu.edu.cn (Z.C.); 2 School of Mathematics and Statistics, Southwest University, Chongqing 400715, China; rsy@swu.edu.cn

**Keywords:** polylactic acid, fiber surface treatment, biocomposites, thermo-mechanical properties, interfacial adhesion

## Abstract

In this study, polylactide acid (PLA) is filled with bamboo fibers (BFs) to fabricate a biodegradable natural composite for industrial applications. The influence of pre-treatment of BFs using 4 wt % sodium hydroxide (NaOH) solution at room temperature for 1 h on thermal and mechanical properties of resultant composites is systematically investigated. Differential scanning calorimetry and thermogravimetric analysis demonstrate that the incorporation of treated BFs promotes higher glass transition and crystallization temperatures of the resultant composites relative to untreated fiber composites, whereas alkali treatment results in superior thermal stability. Furthermore, the fracture surfaces are characterized by scanning electron microscopy. The changes in morphology reveal the possible dissolution of hemicellulose and lignin by alkalization with NaOH, indicative of an improved interfacial adhesion. An increment in the tensile strength of composites is achieved through the reinforcement with treated fibers. However, a lower tensile modulus is found for composites reinforced with chemically modified BFs, which might be due to the partial conversion of cellulose I into II. The results highlight that the use of BFs could be a feasible candidate as reinforcements for the development of biodegradable composites.

## 1. Introduction

Among various biodegradable polymers, PLA has attracted increasing attention as it is possible to achieve high molecular weight with expected lifetime [1]. The excellent mechanical, thermal, and barrier properties and processability of PLA broaden its applications. Besides, PLA can maintain its mechanical properties, even under a humid environment without undergoing rapid hydrolysis. Moreover, it is currently considered amongst the most promising materials in the structural community. Biocomposites that maintain green characteristics mainly consist of bio-based polymers reinforced with natural fibers. Thus, there is an increasing interest in the incorporation of plant fibers extracted from biomass into bioplastics for the development of environmentally friendly composite materials in the field of construction, automobile, and other industries [2,3]. Unfortunately, PLA has many obvious drawbacks when confronted with requirements for certain applications as it exhibits performance deficiencies, such as poor toughness, arising from its inherent brittleness and significantly higher production cost compared to conventional plastics [4]. Therefore, to overcome these drawbacks, some studies reported the use of traditionally synthetic fibers, such as glass fibers, as reinforcements to improve its mechanical properties [5]. However, it is expected that the addition of natural fibers to PLA matrix could reduce the density and lower the cost of products apart from enhancing the performance of virgin material.

It is well known that the major components of lignocellulosic fiber consist of cellulose, hemicellulose, and lignin. Cellulose is a group of microfibrils shaped from d-glucose units connected by β-1,4-glycosidic linkages and controlled between chains by hydrogen bond, providing it highly ordered structure and good properties. Hemicellulose is a group of polysaccharides of pentose and hexose, and plays an important role of supporting cellulose microfibrils. Lignin consists of phenolic constituent with amorphous structures that lead to the stabilization and protection of these fibrils [6,7,8]. Undoubtedly, the great interest toward the plant fibers is related to their physical, chemical, and mechanical properties such as lightweight, cost effective, inexhaustible supply, and biodegradability. Among the well-known cellulose fibers, bamboo is used commonly for the construction of houses, bridges, and home utensils due to its rapid growth rate and universality [9]. Especially in recent years, bamboo has been prominent on the international scene as a sustainable material, being used in the fields of engineering, structural supports and biocomposite design, and incorporated in all production chains. Wang et al. [10,11] reported that these properties of bamboo make it a promising reinforcing material in composites, attributed to the fact that the cellulose fibers in bamboo are aligned along its length, providing maximum tensile strength and rigidity in that direction. Another factor considered decisive in the choice of bamboo as reinforcement material is the high content of cellulose (up to 60%) [12], responsible for the rigidity and resistance [13]. Prasad and Rao confirmed that bamboo fiber (BF)-reinforced composites with varying fiber content possessed superior tensile strength and flexural strength compared to jowar- and sisal-fiber reinforced composites [14].

Despite many excellent properties, one major challenge in using BFs arises from its hydrophilic character. The presence of easily accessible hydroxyl groups within the cellulose structure leads to high moisture absorption, which impedes intimate intermolecular contact with hydrophobic polymer matrices. Low interaction with matrix, consequently, reduces fiber reinforcing efficiency due to poor compatibility between these combined constituents, leading to lack of stress transfer from the matrix to load bearing fibers. Such a shortcoming adversely produces a disastrous consequence for the mechanical properties of composites. Fuentes et al. [15] conducted a study of physical adhesion of BF with thermoplastics by applying the molecular-kinetic theory of wetting. They found that bamboo polyvinylidene-fluoride composites exhibited an improved interfacial strength due to strong physical interactions at the interface, compared to other thermoplastic matrices, such as polypropylene, maleic anhydride grafted polypropylene, and polyethylene terephthalate. It is justified that surface modification using chemical treatment of the cellulose fibers appears a necessity to decrease the polarity of cellulose and in turn promote the effective bonding with resin matrix during the fabrication of composite material [16]. Indeed, alkali treatment is widely used in the stage of surface modification to remove a certain amount of hemicellulose, lignin, pectin, and other non-cellulosic components covering the fiber surface, resulting in better-purified cellulose. Manalo et al. [17] reported that an improvement in mechanical properties of BF-polyester composites was achieved by using NaOH. This was attributed to the removal of hydrophilic components from the fiber, allowing its better compatibility with the matrix. Moreover, such a method can prevent moisture absorption and increase fiber surface roughness available for superior adhesion with the surrounding matrix [18]. Furthermore, Wang et al. performed an experimental investigation for analyzing the effect of alkali treatment on the morphology and properties of BF [9], and further revealed an improvement in fiber tensile strength with the use of 4 wt % NaOH solution [19]. Recently, Qian et al. [20] conducted a study on the mechanical properties and interfacial bonding of PLA-based composites reinforced with bamboo particles, suggesting that low-concentration NaOH solution is beneficial to the fiber pretreatment to reinforce such composites. Although several attempts have been made to explore the influence of alkali treatment on the properties of some natural fiber composites, very limited efforts have been endeavored to the use of BFs as reinforcements for PLA, and little knowledge of the thermo-mechanical performance of such thermoplastic biocomposites has yet been obtained.

In this study, PLA resin matrices were filled with long BFs for the preparation of unidirectional composites. The influence of fiber surface treatment on the properties of the ensuing composites was determined by experimental investigations in terms of morphological characterization as well as thermo-mechanical properties. Scanning electron microscopy (SEM), X-ray diffraction (XRD), differential scanning calorimetry (DSC), thermogravimetry analysis (TG), and tensile tests were carried out to observe the alkali-induced changes on PLA-based composites. This study is expected to provide detailed information to support development and application of a cost-effective and eco-friendly biocomposite through a comprehensive understanding of its thermo-mechanical characteristics. Noteworthy, for analytical purpose, the present study mainly focused on characterizing the thermo-mechanical properties of the PLA-based composites reinforced with BFs with and without alkali treatment; however, other influential factors, such as fiber content, crystalline structure of PLA matrices, and phase structure were not considered. Undeniably, many more systematic explorations are demanded to investigate the effect of these factors on the thermo-mechanical properties of the composites, which will be pursued in our future research.

## 2. Experimental Details

### 2.1. Materials

BFs (about 1.2 g·cm^−3^) were used as the reinforcements in this study. A six-year-old natural bamboo that belongs to one of the most popular bamboo species in China, known as moso bamboo, was obtained from a bamboo plantation located in Fujian Province, China, with the subtropical humid monsoon climate. The fibers supplied from Jian Zhou Group of Fuzhou, China were extracted from the bamboo culms by steam explosion method. Noteworthy, harvesting of culms was mostly carried out during the winter season to avoid borer damage. Furthermore, PLA used as the matrix resin was of grade 3051 D, produced by NatureWorks^®^, Suzhou, China, and it was a continuous filament of chopped staple fiber. The number-average molecular weight (*M*_n_), weight-average molecular weight (*M*_w_), and some other thermo-mechanical characteristics for the PLA sample are listed in Table 1. Chemicals used in the treatment, such as NaOH with 96% purity, were of analytical grade obtained from local commercial sources.

### 2.2. Alkali Treatment

The chemical treatment (alkali solutions) was performed to partially remove the lignin, hemicellulose, and other residues from the fiber surface. The BFs (120–180 μm diameter) were treated with 4 wt % NaOH solution at room temperature for 1 h, maintaining a liquor ratio of 20:1 that represents the ratio of the weight of liquor used to the weight of fibers being treated. Further, the treated fibers were washed several times with distilled water to remove the remaining NaOH from the surface until the water no longer indicated any alkalinity reaction by the evidence of pH = 7. The washed fibers were dried at room temperature for 48 h, followed by oven drying at 80 °C for 2 h [17]. Similarly, the PLA fibers underwent a drying treatment by applying heat (80 °C) under vacuum for 24 h to eliminate the excessive moisture responsible for hydrolysis during sample processing. Finally, BFs and PLA were kept in a desiccator (ZKF040, Shanghai Experiemtal Instrument Factory Co., Ltd., Shanghai, China) with sealed polythene bags to avoid contamination due to atmospheric moisture prior to composite manufacturing.

### 2.3. Preparation of Composite

A biocomposite was produced by hot-pressing pre-forms using a press-molding machine (Y/TD71-45A, Tianduan Press Co., Ltd., Tianjin, China). Prior to composite fabrication by compression molding, BFs were wound and stretched around a metallic plate, and then PLA fibers were uniformly mixed with the BFs to obtain a good combination of BF and PLA. The pre-forms of fibers embedded in resin were dried at 90 °C for 1 h and cut into a stainless steel mold with geometry corresponding to the thickness of 2 mm, the length of 175 mm, and the width of 170 mm. Subsequently, the dried pre-form was placed in the mold and pressed under a pressure of 3 MPa at 150 °C for 4 min, then releasing the pressure for 1 min. The process was repeated two times to eliminate the air bubbles from the composite. Then, kneading was continually conducted on the sample for 20 min at 185 °C under a pressure of 15 MPa. After cooling at the current pressure for 8 h in the machine, the composite plate was then taken off the mold, and then rectangular shaped specimens were achieved through cutting into required dimensions of 150 mm × 20 mm × 2 mm in length, width and thickness, respectively. Following these steps, the samples were appropriately polished with fine sandpaper to avoid flaws that could act as sources for stress concentrations along the edges. To avoid stress concentration, all specimens were equipped with 35 mm long and 2 mm thick aluminum end-tabs, leaving the gauge length of 80 mm. Fiber volume fraction was set around 50% by weight measurements. Notably, the composites were equilibrated in constant relative humidity of 50% before testing. In this study, configuration was limited to unidirectional lamina with a ply angle of 0°, and the length of continuous BFs equaled that of specimen, i.e., 150 mm in case of tensile testing. For the purpose of simplicity, the nomenclature of the composites used in this study is as follows: the abbreviation “UTFC” represents untreated fiber composites, and “ATFC” denotes alkali-treated fiber composites. Moreover, the abbreviation “UTF” represents untreated fibers, and “ATF” denotes alkali-treated fibers.

### 2.4. Test Methods

#### 2.4.1. Tensile Test

The mechanical performance of the composites was evaluated with respect to tensile strength, tensile modulus, and fracture strain. For each composite, five samples were tested for their tensile properties using an electromechanical universal testing machine (CMT4204, Skyan Power Equipment Co., Ltd., Shenzhen, China) fitted with a Celtron 50 kN load cell (model PSD-5tSJTH, serial number 33766) in accordance with ASTM D638-10 [21]. During the tensile test, all specimens were clamped at both ends in the chuck to guarantee the synchronization of the displacement increasing in the fiber and matrix phases. They were held under load until tensile failure occurred, the time-to-failure being defined as the time at which the lamina could no longer support the externally applied load. The tensile strength and modulus were obtained directly from the machine. Noteworthy, all the tensile tests were performed at room temperature of 25 °C under the same humidity, with a crosshead speed of 0.5 mm·min^−1^.

#### 2.4.2. Scanning Electron Microscopy

The morphological characterization of the fracture surface after the tensile test for both untreated and treated composites was investigated by SEM (JELO JMS-6610, Tokyo, Japan) at room temperature operated at a voltage of 10 KV. For SEM observations, all the samples were sputtered with a thin layer of gold with thickness of approximately 10 nm to make them conductive before fractographic inspection.

#### 2.4.3. X-ray Diffraction

The diffraction spectra of UTFC and ATFC were collected using radiation generated by an X-ray diffractometer (XRD-6100, Kyoto, Japan) equipped with Ni-filtered Cu *K*α radiation at room temperature. The diffraction patterns were measured in the angle (2θ) range from 5° to 35° at a scanning rate of 2°·min^−1^. The operating voltage and current were 40 kV and 30 mA, respectively. Furthermore, the crystallinity degree (*X*_c_) of UTF and ATF were determined using an MDI JADE (Materials Data Inc., Livermore, CA, USA), which is a professional analysis system for interpreting the XRD data.

#### 2.4.4. Thermogravimetric Analysis and Its Derivative

The thermal behavior of composite was evaluated by TG, using a synchronous differential thermal analyzer (model STA409PC, NETZSCH, Selb, Germany) on five samples for each material type. The decomposition analysis was performed under nitrogen atmosphere to avoid unwanted oxidation. Each measurement was carried out on around 10 mg of each sample (PLA, BF, UTFC, and ATFC) over a temperature range from ambient to 600 °C at a heating rate of 10 °C·min^−1^.

#### 2.4.5. Differential Scanning Calorimetry Analysis

DSC measurements were performed using a TA Instruments DSC Q20 analyzer (New Castle, DE, USA) with 70 μL aluminum oxide crucible, on five samples for each material type. A sample of about 8 mg was tested under non-isothermal conditions according to the following thermal program: heating from 20 to 220 °C (5 min hold to eliminate the thermal history), cooling to 20 °C, and heating to 220 °C, and all steps were performed at the rate of 10 °C·min^−1^. The measurements were conducted under a nitrogen gas atmosphere at a flow rate of 80 mL·min^−1^. The glass transition temperature (*T*_g_), melting temperature (*T*_m_), cold crystallization temperature (*T*_cc_), enthalpy of fusion (Δ*H*_m_), and enthalpy of cold crystallization (Δ*H*_cc_) were recorded during the second heating process. The crystallinity index of the sample was calculated by using the following Equation (1):

(1) $$X_{c}(\%)=\frac{\Delta H_{m}-\Delta H_{\mathrm{cc}}}{\Delta H_{0}}\times 100,$$

where Δ*H*_0_ is the enthalpy of fusion of perfectly 100% crystalline PLA (J·g^−1^), taken as 93 J·g^−1^ [12]. 

## 3. Results and Discussion

### 3.1. Mechanical Properties

**Figure 1:**
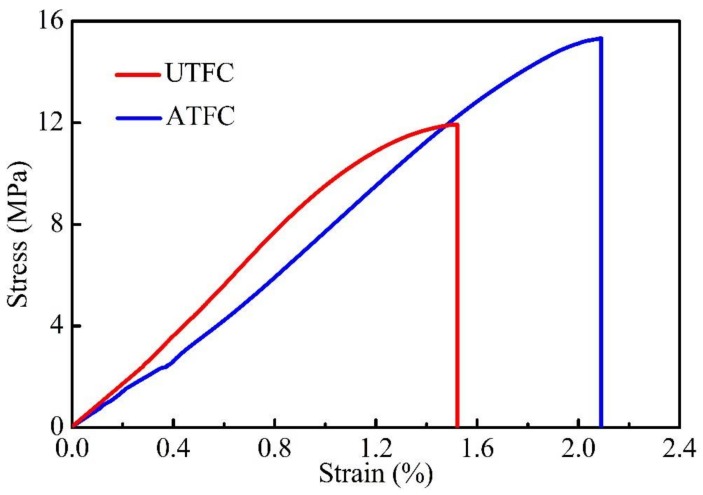
Typical stress-strain curves of some composite specimens from tensile tests.

Figure 1 shows the typical stress-strain relationship obtained from the tensile tests. Clearly, the stress-strain curve is divided into approximately two regions. The first region up to 0.3% strain is characteristic of an almost linear behavior, allowing for the measurement of the modulus. In the second region, the irregular curve demonstrates a nonlinear behavior, which is probably due to the occurrence and accumulation of other potential damage except fiber breakage [11]. The slope of the diagram decreases gradually compared to the region I. When the load reaches the ultimate point, the curve is followed by a sudden drop and the elongation at break could be obtained accordingly. The strain to failure for all tested composites is less than 5%, which indicates the occurrence of a brittle failure. Moreover, the areas under the curves corresponding to the untreated and treated fiber composites are 10.45 and 16.85, respectively, which confirms that the toughness of the composite increases after fiber surface treatment [22]. 

The tensile properties of BF/PLA composites obtained from the tests are listed in Table 2. Interestingly, the tensile strength of composites increases by 49% and elongation at break by 84%, while the tensile modulus decreases by 21% after fiber surface modification. The table summarizes that after the ANOVA (Analysis of Variance) test, a significant difference is observed for the testing groups. This indicates that the treatment has an important effect on the mechanical properties of composites. 

**Figure 2:**
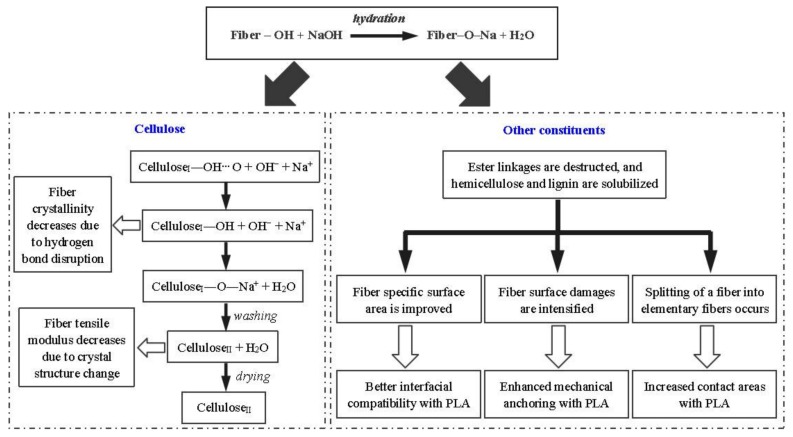
Reaction mechanism of alkali treatment.

**Figure 3:**
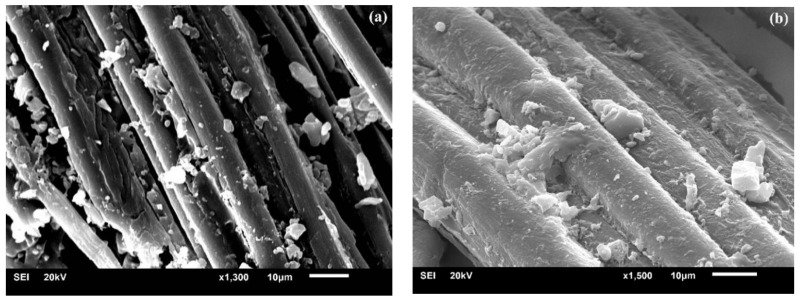
SEM images of surface morphology of composites: (**a**) before alkali treatment; and (**b**) after alkali treatment.

The effect of alkali treatment on cellulose fiber is presented in Figure 2: First, Na^+^ and OH^−^ are produced during the hydration reaction, and then the hydroxide ions enter into the gap between the cellulose layers and destroy the hydrogen bonds within the cellulose molecules, which contributes to the increment of active OH-groups and further increase the amorphous area. In this case, the OH-groups of cellulose in the lattice are converted into ONa-groups and form new Na-cellulose I lattice. This has also been proven in our previous investigation on BFs [19]. The disruption of hydrogen bond leads to the dissolution of cellulose. Consequently, many molecular chains get separated, resulting in lower polymerization degree. Finally, the original parallel-chain crystal structure of cellulose I gets converted to more stable cellulose II by recrystallization of the chains upon washing and drying, ultimately altering the crystallinity, accessibility, and orientation of cellulosic fiber [23,24]. Such a transition phenomenon is also termed as mercerization, leading to fibrillation by removing cementing materials, and it contributes to reduce the fiber diameter and increase its aspect ratio. This consequently leads to the splitting of BF into many elementary fibers, as reported in our previous study [19,25]. Qian et al. [20] claimed that alkali treatment increased the specific surface area of BFs, which could lead to a higher probability of PLA penetration into the fiber surface. Figure 3 displays the elementary fibers embedded in UTFC and ATFC. Figure 3a exhibits that the elementary fiber surface (without alkali treatment) is rarely covered with PLA, indicative of a weak bonding between elementary fiber and PLA. In contrast, PLA infiltrates the gaps of cellulose fibers after alkali treatment, indicating strong linkage between these elementary fibers [26]. 

**Figure 4:**
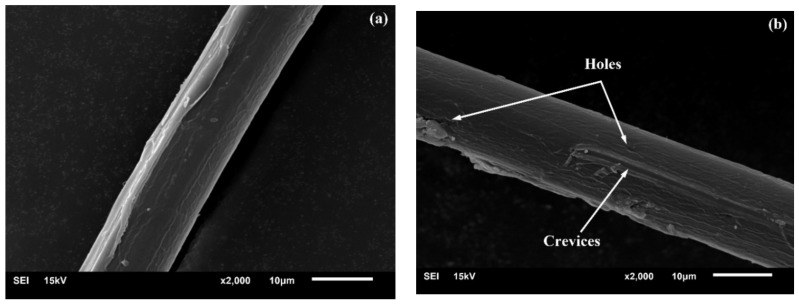
SEM images of surface morphology of an elementary fiber: (**a**) before alkali treatment; and (**b**) after alkali treatment.

**Figure 5:**
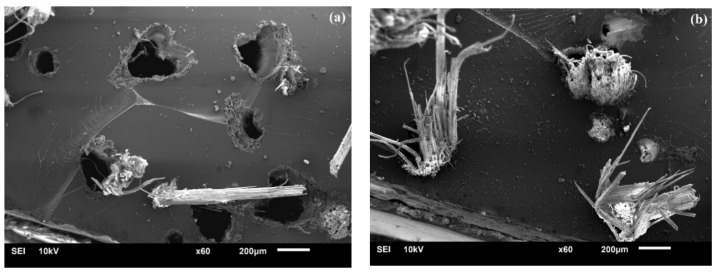
SEM images of tensile fracture surface of: (**a**) UTFC; and (**b**) ATFC.

Figure 4 clearly demonstrates that the impurities dissolved from the elementary fiber surface by the alkali treatment increase its roughness [27,28]. Moreover, the presence of some holes and crevices exposed on its surface contributes to the formation of mechanical anchoring with the matrix, leading to a better fiber-matrix adhesion [4,29]. The above-mentioned analysis concludes that the fibrillation leads to an increase in the effective surface area available for contact with the matrix and such a behavior improves superior interfacial bonding as well as tensile strength. This could be evidenced by typical SEM images shown in Figure 5, which clearly display the occurrence of more fiber pull-out in UTFC. 

**Figure 6:**
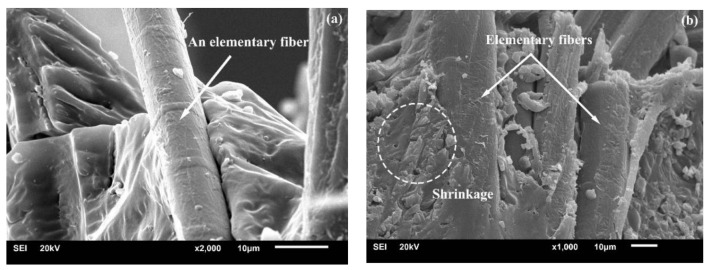
SEM images of tensile fracture morphology of: (**a**) UTFC; and (**b**) ATFC.

Furthermore, Figure 6 illustrates the interfacial adhesion in composites with and without treatment when fracture occurs. Figure 6a shows the elementary fiber pull-out, indicating the poor interface adhesion in UTFC system. In contrast, Figure 6b shows the difference in the interfacial adhesion quality, indicative of the fact that the broken elementary fibers are still covered by the resin in ATFC system. Moreover, the surrounding PLA matrix at the interface shows more pronounced shrinkage, which indicates that a larger force should be applied to pull out the fibers from the matrix [30]. These findings could prove a positive effect of fiber surface modification on the interfacial bonding, thus the incorporation of treated BFs into neat PLA could help in improving the tensile strength of matrix by about 5.1 MPa.

Table 3 summarizes that the crystallinity degree of BFs is discovered to decrease after fiber surface modification. Combined with the above-mentioned reaction mechanism, the role of the alkali treatment is considered to disrupt the hydrogen bonding of cellulose and consequently increase the amorphous area at the expense of crystallinity [4]. Previous FTIR results [9] show the partial removal of the acid groups and ester groups of hemicellulose, pectin, and lignin from the fiber surface during alkali treatment. Indeed, the presence of hemicellulose and lignin between the cellulose microfibrils allows themselves to get fixed in a position to prevent the occurrence of slipping [31]. Thus, the decrease of crystallinity and orientation degree of cellulose crystals along the fiber axis leads to the loosening of the fiber structure so that the elongation at break increases [32]. The Young’s modulus of cellulose II is lower than that of cellulose I [33], and it is suggested that cellulose II deforms much more than cellulose I. Thus, the partial conversion of cellulose I into cellulose II is the reason that a higher tensile modulus is obtained for UTFC compared to that for ATFC [34].

### 3.2. Thermal Properties

**Figure 7:**
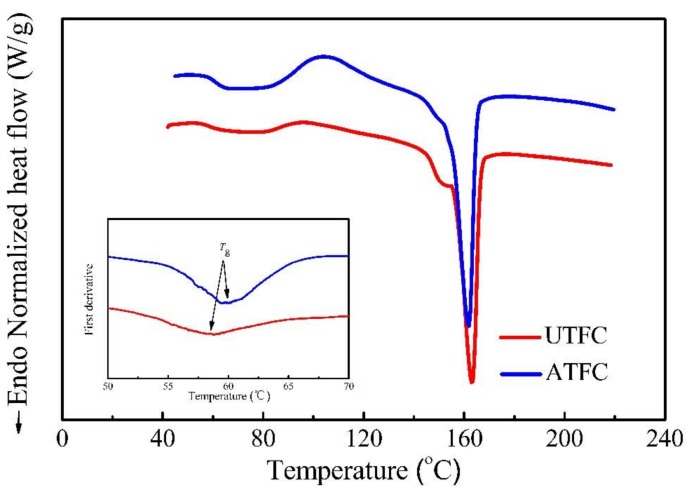
DSC curves of PLA-based composites reinforced with untreated and treated BFs.

Figure 7 shows the DSC trace of composites with treated and untreated BFs, where *T*_g_, *T*_cc_, and *T*_m_ could be determined from the heating scan. Noteworthy, the insert is the plot of the first derivation of DSC in the temperature range of 50 to 70 °C, and in turn *T*_g_ could be achieved through the extreme point. The peak of cold crystallization represents recrystallization of imperfect crystals during the second heating process, and it appears distinctly in the curve of ATFC. Indeed, the larger peak area in the diagram represents an increase in the quantity of these crystals in ATFC, indicating that such a composite is poorly crystalline [34]. In the second endothermic stage, two apparent melting peaks are observed in the thermogram of UTFC, which could be attributed to the transition of melt/recrystallization mechanism of PLA crystals [35]. The thermal characteristics obtained from DSC studies are summarized in Table 4. In the case of ATFC, the non-crystalline molecular chains of PLA are severely restricted by being anchored to the immobile crystallites [36,37]. This causes the declined mobility of the matrix chains, leading to a higher *T*_g_. This phenomenon is an indication of a better interfacial adhesion achieved by treatment. Furthermore, the shifting of *T*_cc_ to a higher temperature in ATFC is associated with the worse nucleating effect of the treated fibers that negatively influence the PLA crystallization. For ATFC, the presence of the single melting peak indicates that considerable disordered crystals could be shaped, leading to a lower *T*_m_. Accordingly, the molecular chains are tightly constrained by the strong interface that needs more energy to regain arrangement of PLA, which results in an improvement of Δ*H*_cc_. The degree of crystallization (*X*_c_) of ATFC (9.7%) is lower than that of UTFC (21.2%), and it decreases by 54%. This may be attributed to the decrease in the crystal area and increase in the amorphous region by alkaline solution, which is confirmed by the change in Δ*H*_cc_. However, Qian et al. [20] found that the alkali-treated bamboo particles acted as plasticizer or impurity and exhibited a better nucleating effect on the PLA crystallization, which could inversely lead to the increment in crystallinity. The comprehensive analysis indicates that fiber surface modification affects the thermal behavior.

**Figure 8:**
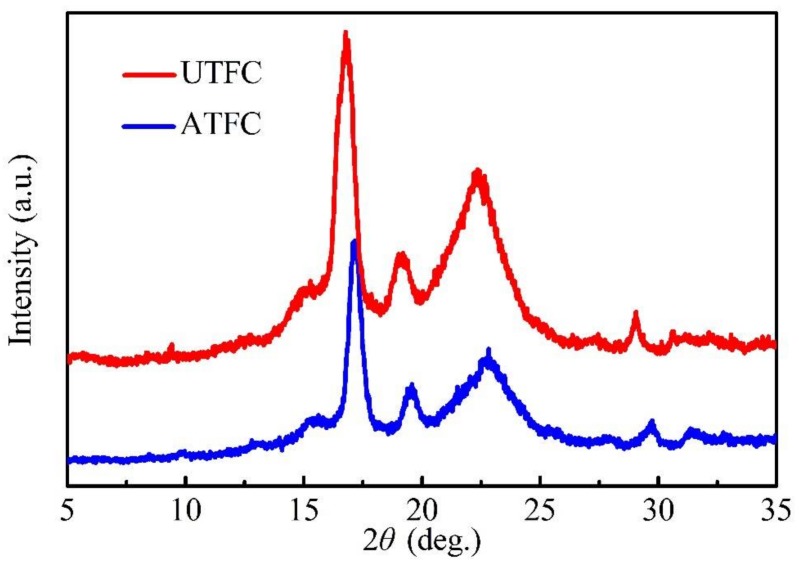
XRD patterns of PLA-based composites reinforced with untreated and treated BFs.

The main objective of the XRD test was to better understand the effect with respect to molecular structure of alkali-treated composites. Figure 8 shows the diffraction patterns of UTFC and ATFC, which evidently exhibit three main peaks. The spectrum of UTFC shows the first sharp peak at 2θ = 16.8° and the second peak at 19.2°, which are characteristic of PLA. As some researchers argued that the peak at 22° might be the result of an overlap of two diffraction peaks of cellulose and PLA [19,38], the third peak at 22.3° is most likely to cover the characteristics of these two constituents. Compared to UTFC, the first peak of ATFC exhibits a slight shift to the higher angle of 17.1°. This could be explained by the fact that the lattice spacing of PLA crystal decreased by alkali treatment, leading to a more compact arrangement of its molecular chains [39]. Evidently, the height of the first peak of ATFC decreases in contrast to that of UTFC. In other words, ATFC exhibits lower peak intensity, which reveals the reduction in the composite crystallinity due to alkali action [40]. This conclusion agrees with the results of DSC.

**Figure 9:**
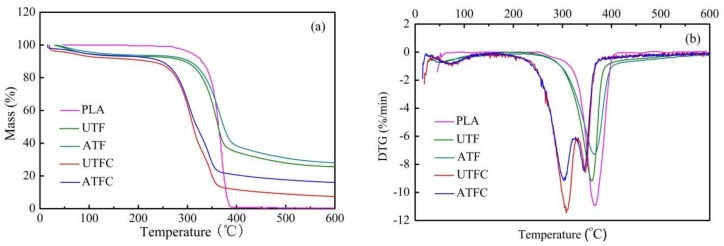
(**a**) TG; and (**b**) DTG curves of PLA, BF, and composites reinforced with untreated and treated BFs.

TG helps to determinate the degradation behavior of in the components that make up a composite, which is based on the measurement of weight change related to temperature. Besides, the magnitude and location of peaks found in the derivative thermogravimetric (DTG) curve provide information about the components of the composites and their interactions on the temperature scale [41]. TG and DTG curves of PLA, UTF, ATF, UTFC and ATFC are presented in Figure 9. The peak temperatures and the corresponding weight loss percentage obtained from DTG curves are summarized in Table 5. Figure 9a shows the TG thermogram, demonstrating the complete thermal degradation of PLA in a single stage at 373 and 402 °C. Previous study [9] on BFs reveals that weight loss in the temperature range from 200 to 330 °C is caused by the degradation of hemicellulose. Further, the weight loss occurring in the temperature range of 330 to 356 °C is derived from the decomposition of cellulose. The decomposition process, appearing at higher temperatures in the range of 356 and 450 °C, occurs mainly on the lignin. The PLA/BF composites degrade through the three main stages, the profiles of which are determined by individual thermal degradation behaviors of BF and PLA. The first step initiates around 70 °C and no significant weight loss is observed in the temperature range of 70 to 200 °C, which is attributed to the evaporation of water combined with hydroxyl groups that are present in the cellulose structure. In the following step, the temperatures are concentrated in the range of 200 to 330 °C, and the weight loss is mainly caused by pyrolysis of cellulose and hemicellulose [19]. In this stage, the decomposition rate of the sample reaches the maximum as shown in Figure 9b. As a result, these changes could increase the effective contact area available for wetting by the matrix resin and improve the interfacial adhesion between the fibers and matrix. The third transition occurs above 330 °C, and decomposition occurs with a lower rate of the weight loss. The peak at approximately 346 °C is due to the decomposition of the lignin and PLA macromolecular chains [19]. In the third stage, all the volatile materials are disposed from the sample, thus the residual mass remains unchanged. When the temperature is above 450 °C, the weight loss of UTF, ATF, and PLA is 69.3%, 66.4%, and 99.5%, respectively, which indicates that the decomposition of PLA is responsible for the weight loss of the composite. Evidently, the composites exhibit a lower degradation temperature than that of PLA, which is attributed to the decrease of molecular weight of PLA at high processing temperature (185 °C) [42]. Thus, the results indicate that the addition of BF to PLA matrix significantly affects the thermal degradation temperature. The table also shows that the surface treatment decreases the percent of weight loss, leading to better thermal stability of the composites. These effects are consistent with the literature studies, reporting that better penetration of PLA into fibers after treatment is responsible for thermal stability [43,44]. In the third stage, the peak temperatures of UTFC and ATFC are found to be close to the degradation peak of lignin.

## 4. Conclusions

In this study, bamboo fibers (BFs) are chemically modified by alkali treatment and its effects on the properties of ensuing PLA-based composites are also investigated through different experimental approaches. The mechanical tests for tensile properties reveal the substantial increase in the tensile strength and elongation at break of ATFC by 49% and 84%, respectively, compared to those of UTFC. This could be ascribed to the formation of an effective adhesion allowing good impregnation of the fibers by matrix after treatment. However, the modulus loss might be attributed to the partial conversion of cellulose I into II. SEM images further confirmed the achievement of superior interfacial adhesion by fiber surface modification, which is found to be beneficial to the load transfer between BF and PLA, leading to an improvement of tensile strength. DSC reveals that the treated fibers acted as nucleating agent and negatively affected the polymer crystallization, which is also confirmed by XRD. However, TG tests indicate that PLA could better penetrate the treated fiber thereby promoting a better interface compatibility, resulting in superior thermal stability of ATFC. The results also provide a possibility of fabricating a long fiber composite by the manufacturing approach for a specific application.

As a final remark, it should be emphasized that the positive information presented herein intensifies the understanding of thermo-mechanical properties of PLA-based composites reinforced with BFs. However, physical properties of PLA polymers rely on their molecular characteristics as well as ordered structures such as crystallinity, morphology and degree of chain orientation [45,46]. Consequently, the properties of such composites are not only dominated by the interfacial interactions, but also affected by the phase structure, crystalline structure of polymer, and fiber content as well [47]. In other words, consideration of the effect of interfacial bonding between fiber and matrix alone is insufficient for determining the properties of composites. A more comprehensive investigation, capable of explaining such effects as fiber arrays, fiber dispersion and structural characteristics of polylactide matrices on composite performance, also demands further systematic explorations in future research.

## Figures and Tables

**Table 1:** Molecular weight and thermo-mechanical properties of PLA sample.

*M*_n_ (kg/mol)	*M*_W_ (kg/mol)	*M*_W_/*M*_n_	*T*_g_ (°C)	*T*_c__c_ (°C)	*T*_m_ (°C)	L/D	Diameter (μm)	*ρ_m_* (g/cm^3^)	Tensile Strength (MPa)	Tensile Modulus (MPa)	Elongation at Breaks (%)
23.5	40	1.7	48.0	83.2	159.6	98:2	12.64	1.26	10.9	411.5	5.2

**Table 2:** Mechanical properties of composites.

Performance	Average Value for UTFC	Average Value for ATFC	*p* Value (*t*-Test) for UTFC and ATFC (α = 0.05)	Significant Difference
Tensile strength (MPa)	10.7 ± 1.37	16.0 ± 0.60	0.004	Yes
Elongation at break (%)	1.3 ± 0.15	2.4 ± 0.24	0.003	Yes
Tensile modulus (MPa)	745.8 ± 70.33	586.8 ± 23.89	0.021	Yes

Note: Two-sided *t*-test in ANOVA, and α is a significant level. For *p* ≥ 0.05, there is no significant difference.

**Table 3:** Crystallinity degree of BFs.

Performance	Average Value for UTF	Average Value for ATF	*p* Value (*t*-Test) for UTF and ATF (α = 0.05)	Significant Difference
*X*_c_ (%)	43.5 ± 1.04	35.7 ± 2.81	0.011	Yes

Note: Two-sided *t*-test in ANOVA, and α is a significant level. For *p* ≥ 0.05, there is no significant difference.

**Table 4:** Thermal characteristics of composites by DSC.

Performance	Average Value for UTFC	Average Value for ATFC	*p* Value (*t*-Test) for UTFC and ATFC (α = 0.05)	Significant Difference
*T*_g_ (°C)	57.3 ± 1.5	59.9 ± 0.7	0.048	Yes
*T*_cc_ (°C)	93.7 ± 1.9	104.8 ± 1.6	0.002	Yes
*T*_m_ (°C)	161.9 ± 1.3	154.4 ± 3.4	0.025	Yes
Δ*H*_cc_ (J/g)	3.4 ± 2.0	9.6 ± 2.3	0.025	Yes
Δ*H*_m_ (J/g)	19.3 ± 2.3	16.0 ± 2.0	0.133	No
*X*_c_ (%)	21.2 ± 1.4	9.7 ± 2.5	0.002	Yes

Note: Two-sided *t*-test in ANOVA, and α is a significant level. For *p* ≥ 0.05, there is no significant difference.

**Table 5:** Thermal properties of composites obtained from TG/DTG.

Performance		Average Value for UTFC	Average Value for ATFC	*p* Value (*t*-Test) for UTFC and ATFC (α = 0.05)	Significant Difference
Peak temperatures (°C)	I	75.7 ± 1.4	73.7 ± 2.1	0.252	No
	II	302.1 ± 2.5	292.4 ± 5.3	0.045	Yes
	III	357.6 ± 2.7	346.5 ± 1.0	0.002	Yes
Weight loss (%)	I	5.3 ± 0.5	3.2 ± 1.1	0.038	Yes
	II	44.2 ± 1.3	34.4 ± 3.1	0.007	Yes
	III	76.3 ± 1.1	68.1 ± 1.0	0.001	Yes

Note: Two-sided *t*-test in ANOVA, and α is a significant level. For *p* ≥ 0.05, there is no significant difference.
